# Binge-Like Eating Is Not Influenced by the Murine Model of *OPRM1* A118G Polymorphism

**DOI:** 10.3389/fpsyg.2019.00246

**Published:** 2019-02-11

**Authors:** Bryn L. Y. Sachdeo, Lei Yu, Gina M. Giunta, Nicholas T. Bello

**Affiliations:** ^1^ Nutritional Sciences Graduate Program, School of Environmental and Biological Sciences, Rutgers, The State University of New Jersey, New Brunswick, NJ, United States; ^2^ Department of Genetics, School of Arts and Sciences, and Center of Alcohol Studies, Graduate School of Applied and Professional Psychology, Rutgers, The State University of New Jersey, New Brunswick, NJ, United States; ^3^ Department of Animal Sciences, School of Environmental and Biological Sciences, Rutgers, The State University of New Jersey, New Brunswick, NJ, United States

**Keywords:** rs1799971, mu opioid, VYVANSE, MOPR, loss of control eating

## Abstract

Impairments in opioid receptor signaling have been implicated in disordered eating. A functional variant of the *OPRM1* gene is a guanine (G) substitution for adenine (A) at the 118 position of exon 1 (A118G). The influence of the A118G variant on binge eating behaviors and the effectiveness of pharmacotherapies used to treat binge eating have not been characterized. Mice were generated with A to G substitution at the 112 position on exon 1 to produce a murine equivalent of the human A118G variant. Homozygous female mice (AA or GG) were exposed to intermittent access to a highly palatable sweet-fat food with or without prior calorie deprivation to promote dietary-induced binge eating. There were no genotype-dependent differences in the dietary-induced binge eating. However, GG mice exposed to intermittent calorie restriction (Restrict) had higher body weights compared with GG mice exposed to intermittent sweet fat-food (Binge) and *ad libitum* feeding (Naive). Acute oral dosing of lisdexamfetamine (0.15, 0.5, and 1.5 mg/kg) or sibutramine (0.3, 1, and 3 mg/kg) did not produce genotype-dependent differences in binge-like eating. In addition, no genotype-dependent differences in binge-like eating were observed with chronic (14-day) dosing of lisdexamfetamine (1.5 mg/kg/day) or sibutramine (3 mg/kg/day). In the chronic dosing, body weights were higher in the GG Restrict compared with AA Restrict. Our findings suggest that the A112G polymorphism does not influence binge eating behaviors or pharmacotherapies for treating binge eating.

## Introduction

Eating disorders, such as bulimia nervosa (BN), binge eating disorder (BED), and anorexia nervosa (AN), are psychiatric illnesses that are moderately influenced by genetic factors (Hinney and Volckmar, 2013; Juli and Juli, 2014). Twin and family studies have indicated heritability estimates to be 62% for BN, 39% for BED, and 34.9% for AN (Javaras et al., 2008; Bulik et al., 2010; Pettersson et al., 2018). Similar to studies of other psychiatric disorders, large-scale genetic studies examining data gathered from single nucleotide polymorphism (SNP) arrays have been used to determine common genetic variation within eating disorder populations (Hubel et al., 2018; Pettersson et al., 2018). However, one missing element in understanding the genetic influences on eating disorders is determining the impact of SNPs on eating disorder traits.

One common nonsynonymous SNP is that of the mu-opioid receptor (*OPRM1*), such that a guanine (G) is substituted for adenine (A) in the 118 position on exon 1 of OPRM1 gene (*A118G OPRM1*; *rs1799971*). This A to G nucleotide substitution changes a putative *N*-glycosylation sequence on the mu-opioid receptor protein from an asparagine to an aspartic acid amino acid (N40D) (Bond et al., 1998; Shi et al., 2002). The N40D substitution has been demonstrated to decrease the half-life of the mu-opioid receptor, which decreases protein stability (Huang et al., 2012). The A118G polymorphism has been estimated to occur in ~11% of Caucasian populations and as high as ~52% in some Asian populations (Bergen et al., 1997; Bond et al., 1998; Tan et al., 2003). Several studies have suggested that the *A118G OPRM1* variant has increased association with opioid use, alcohol dependence, and pain modulation (Bond et al., 1998; Tan et al., 2003; Kim et al., 2004; Bart et al., 2005; Pecina et al., 2015). This *A118G OPRM1* polymorphism also has been found at higher frequency in individuals diagnosed with BED in an obese population (*n* = 136) (Davis et al., 2009). In fact, the BED group had a G allele frequency of 18.5% compared with 9.6% of those obese individuals without BED. In addition, the subjects with BED were also more responsive to the hedonic properties of food (Davis et al., 2009). Despite these findings, the role of the *A118G OPRM1* SNP in binge eating has not been further investigated. Understanding the role of A118G OPRM1 in binge eating could possibly influence the therapeutic options for clinically managing eating disorders. While cognitive behavioral therapy (CBT) and intrapersonal therapy (IPT) are considered standard effective therapies for eating disorders, pharmacotherapy is now emerging as a clinical option (de Jong et al., 2016; Bello and Yeomans, 2018). Sibutramine, a monoamine reuptake inhibitor, was shown to be effective in reducing binge eating behaviors in BED patients in a 24-week randomized placebo-controlled trial (Wilfley et al., 2008). Sibutramine, however, was withdrawn from the US, Australian, and European markets in 2010 in response to treatment-emergent cardiovascular risks (Finer and Executive Steering Committee of the Sibutramine Cardiovascular Outcome, 2010; James et al., 2010; Williams, 2010). Currently, lisdexamfetamine is the only pharmacotherapy approved by the FDA for the maintenance of moderate to severe BED on the US market (Shire, 2015). Lisdexamfetamine dimesylate is a prodrug that has l-lysine conjugated to dextroamphetamine and has been demonstrated in randomized placebo-controlled trials to effectively reduce the frequency, decrease the severity, and attenuate the 30-day relapse rates of binge eating in patients with BED (McElroy et al., 2015; Gasior et al., 2017; Hudson et al., 2017). Thus, understanding the interaction of the A118G polymorphism and lisdexamfetamine could potentially improve how this medication is prescribed and help identify potential responders and non-responders within BED populations.

The single-nucleotide substitution of adenine (A) to guanine (G) in the mouse homologue of the human mu-opioid receptor (MOR) gene occurs at the 112 position on exon 1 (Mague et al., 2009). This A112G substitution results in the same amino acid substitution of asparagine (N) to aspartate (D) at position 38, the equivalent of position 40 in the human peptide, similarly eliminating the putative *N*-linked glycosylation site (Mague et al., 2009; Wang et al., 2012; Zhang et al., 2015). Recently, we developed a murine model with an A112G substitution on a C57BL/6 background, and these mice were used for the present experiments. Using a previously described dietary-induced binge eating model, which employs repeated limited access to highly palatable foods (Bello et al., 2014a,b), we examined whether the murine equivalent to the *A118G* variant impacts binge-like eating and whether lisdexamfetamine has a genotype-dependent influence on binge eating behaviors. For these experiments, the effects of lisdexamfetamine will be compared with sibutramine, a monoamine reuptake inhibitor that was effective for reducing binge eating in BED populations (Wilfley et al., 2008).

## Materials and Methods

### Mice

A transgenic mouse model of the *OPRM1 A118G SNP* on a C57BL/6 background was generated by targeted homologous recombination into murine embryonic stem cells (ES cells) (Caliper Discovery and Alliance Services, Hanover, MD, USA). Specifically, the mouse chromosome 10 sequence (n.t.# 3,510,000,000~3,590,000) was retrieved from the Ensembl database and used as a reference in this project. The bacterial artificial chromosome (BAC; RP23-263A7) was used for generating the homology arms and the conditional KO region for the gene targeting vector, as well as the southern probes for screening targeted events. The 5′ homology arm (5.3 kb), 3′ homology arm (3.0 kb), and conditional KO region (2.2 kb) were generated by PCR using high-fidelity Taq DNA polymerase. The fragments were cloned in the pCR4.0 vector and were confirmed by restriction digestion and end-sequencing. The adenine to guanine mutation in exon 1 was introduced into the conditional KO region by PCR-based site-directed mutagenesis with the QuickChange II kit (Stratagene, La Jolla, CA, USA). The final vector obtained by standard molecular cloning contained LoxP sequences flanking the conditional KO region (2.2 kb), Frt sequences flanking the Neo expression cassette (for positive selection of the ES cells), and a DTA expression cassette (for negative selection of the ES cells). The final vector was confirmed by both restriction digestion and end sequencing analysis. *NotI* was used for linearizing the final vector prior to electroporation into C57BL/6 ES cells. Heterozygous mice were obtained from the male chimera breeding to C57BL/6 wild-type females. After subsequent generations, heterozygous mice were bred to ACTBFLPe mice (cat # 005703, The Jackson Laboratory, Bar Harbor, ME, USA) for deletion of the Frt-flanked Neo cassette, and offspring from several generations (>3) was used as founder mice for the *OPRM1 A112G* mouse colony.

The *OPRM1 A112G* mice were maintained by heterozygous breeding. Breeding pairs were placed on a 12:12 h light/dark cycle with lights off at 1,900 h. Pups were kept with the dam until weaning at postnatal day (PND) 21. Genomic DNA from ear clippings was used to genotype animals. Primer sequences are 5′-GCACACAAAAGAGCAATAGAACGGAAATA-3′ and 5′-GATCCCCTCAGAAGAACTCGT-3′. After weaning, female juveniles were group housed. Mice were fed standard chow (Laboratory Rodent Diet 5001, 13.38% fat, 28.67% protein, 3.36 Kcal/g), and water was available at all times. All feeding protocols began at 6 weeks of age in homozygous (AA or GG) female littermate mice. Due to the unequal size of the litters and the need to control for the individual feeding groups, mice were single housed throughout the feeding conditions and experimental dosing periods. This study was conducted in accordance with NIH guidelines. The animal care protocol was approved by the Institutional Animal Care and Use Committee of Rutgers University (OLAW #A3262-01, protocol #13-001).

### Feeding Protocols and Dietary-Induced Binge Eating

At PND 42, female mice (*n* = 254) underwent a 24-h pre-exposure to a highly palatable binge-like food (sweetened fat; hydrogenated vegetable shortening +10% sucrose; 8.6 Kcal/g) and standard chow. One week following the 24-h pre-exposure, mice (PND 49) were randomly assigned to one of four groups controlling for body weight and pre-exposure intake. Feeding groups were based on two independent dietary variables, intermittent calorie deprivation (24 h), and/or intermittent sweetened fat access (30 min). For the 30 min intakes, pre-weighed sweetened fat and/or pre-weighed standard chow were individually placed in jars (5.08 cm diameter × 2.56 cm height). After 30 min, the remaining chow and/or sweetened fat jars were weighed again to determine the intake in grams. The exposure to intermittent calorie deprivation occurred on *days 2* and *5*, while the refeeding with standard chow and 30-min access to the sweetened fat (i.e., “Binge”) occurred on *days 3* and *6* of the 7-day feeding schedule. The 30-min access provides a more robust feeding bout, compared with longer access periods of sweetened fat (e.g., 2 h) (Bello et al., 2009, 2011, 2014a,b). Calorie deprivation and refeeding were at 1,700 h (2 h before lights out at 1,900 h). In this fashion, the *Restrict Binge* group was exposed to a repeated cycle that consisted of three no restriction days (*days 1, 4,* and *7*), two weekly episodes of calorie restriction (*days 2* and *5*), and two weekly episodes of scheduled refeeding starting with 30-min access to an optional highly palatable food (*days 3* and *6*). The second group, the *Binge* group, had *ad libitum* standard chow in addition to the 30-min access to the sweetened fat (*days 3* and *6*) at the same time and frequency as the Restrict Binge group. A third group, *Restrict* group, had an identical pattern of calorie deprivation with standard chow (*days 2* and *5*) as the *Restrict Binge* group but did not have repeated access to the sweetened fat upon refeeding on *days 3* and *6*. A *Naive* group had *ad libitum* standard chow with no access to the sweetened fat. These feeding protocols are modified from a previously published procedure for a rodent model of dietary-induced binge eating (Bello et al., 2011, 2012, 2014a,b), see Table 1. Cumulative calorie intakes and body weight were measured twice a week at the time mice were removed from their cage for the vaginal cytology procedure (09 00 h).

**Table 1:** Feeding groups for the dietary-induced binge eating protocol.

Groups	Calorie restriction (*days 2* and *5*)	Sweetened fat access (*days 3* and *6*)
Restrict binge	Intermittent (24 h, twice a week)	Intermittent (30 min, twice a week)
Binge	None	Intermittent (30 min, twice a week)
Restrict	Intermittent (24 h, twice a week)	None
Naive	None	None

### Vaginal Cytology

Vaginal cytology was performed to determine the stage of estrous. On *days 3* and *6* of the weekly schedule (i.e., refeeding days or “binge days”), vaginal cytology was performed 8 h before the scheduled feeding. Briefly, the vaginal cavities of mice were lavaged with sterile saline (0.9%), and the cells were characterized by vaginal epithelial cell morphology. Proestrus/Estrus was classified by the presence and relative number of nucleated epithelial and cornified cells. Metestrus/Diestrus was classified by the presence and relative number of leukocytes.

### Acute Dosing

After 6 weeks of the feeding protocol, one cohort of mice underwent the within subject crossover scheme of acute dosing. Mice were orally dosed with single-use, sterile plastic feeding tubes (20 ga × 30 mm; cat # FTP-20-30, Instech Laboratories, Plymouth Meeting, PA, USA). Each mouse was orally dosed with vehicle (deionized water); 0.15, 0.5, and 1.5 mg/kg lisdexamfetamine dimesylate (lot # AF7299B; Shire Pharmaceuticals, Lexington, MA, USA); and 0.3, 1, and 3 mg/kg of sibutramine hydrochloride monohydrate (cat # S9944; Sigma-Aldrich, St. Louis, MO, USA). Dosing was performed 60 min before the scheduled refeeding bout on *day 6*. Each mouse received all doses once, and oral dosing was only performed once a week. Mice continued their respective feeding protocol throughout.

### Chronic Dosing

After 6 weeks of the feeding protocol, another cohort of mice underwent a long-term daily dosing regimen. Separate groups of mice received daily oral dosing of vehicle, lisdexamfetamine (1.5 mg/kg), or sibutramine (3 mg/kg) for 14 days. Mice were weighed daily at 0800–0815 h. Dosing was performed 0900–1,100 h daily. Mice continued their respective feeding protocol throughout.

### Statistical Analyses

For the 24-h pre-exposure, the genotype effect on body weight and intakes was analyzed by individual independent *t* test for each variable. For the dietary-induced binge eating, total calorie intakes for 30 min, cumulative intakes, and body weights were analyzed by individual two-way ANOVAs with repeated measures performed to determine the effects of feeding groups, genotype, and interaction. Individual feeding groups were analyzed using ANOVAs with repeated measures. For the within design for the repeated acute dosing, two-way ANOVAs with repeated measures were performed to determine the effects of genotype, feeding groups, and interaction. For the chronic dosing, multivariate ANOVAs with repeated measures were used to determine the influence of genotype, feeding group, treatment, and interactions. Individual two-way ANOVAs with repeated measures were performed on individual feeding groups to determine the effect of genotype, treatment (doses), and interaction. *Post hoc* comparisons were made when appropriate with Newman-Keuls test. All statistical analyses were performed with Statistica 7.1 software (StatSoft Inc.), and significance was set at *α*  =  0.05.

## Results

### Pre-exposure Intakes and Body Weights 7 Days Prior to Starting the Feeding Protocols

At PND 42, body weights were 17.64 ± 0.12 g for AA mice and 17.83 ± 0.15 g for GG mice. The 24-h sweetened fat intakes were 14.56 ± 0.29 Kcal and 15.36 ± 0.30 Kcal for AA and GG, respectively. The 24-h chow intakes were 2.30 ± 0.55 Kcal and 2.09 ± 0.39 Kcal for AA and GG mice, respectively. There were no genotype differences in body weights, sweetened fat, or chow during the 24-h pre-exposure.

### Calorie Intakes During the 30-Min Access Period on Days 3 and 6 Over the 6-Week Feeding Protocols

**Figure 1:**
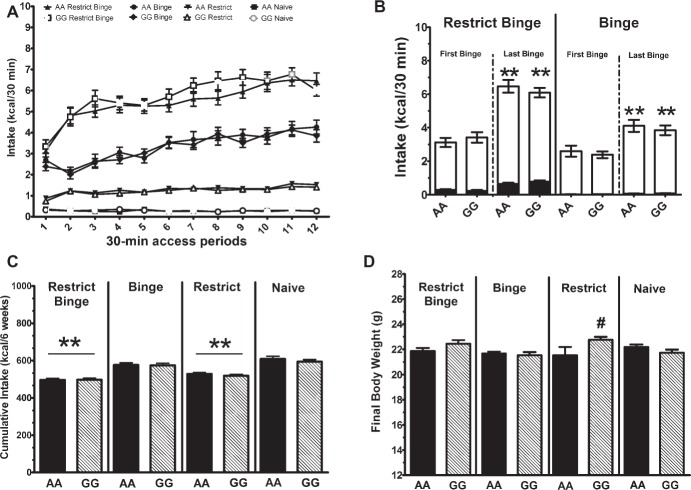
Dietary-induced binge eating protocol over 6 weeks. Data are expressed as mean ± SEM. Mice were exposed to 12 intermittent 30-min eating bouts twice a week. Feeding groups are illustrated in Table 1. The Restrict Binge group (*n* = 31–32/genotype) received intermittent calorie deprivation (24 h) and 30-min access to “sweetened fat” (vegetable shortening +10% sucrose; 8.6 Kcal/g), Binge group (*n* = 31–32/genotype) received intermittent 30-min access to sweetened fat, Restrict group (*n* = 32/genotype) received intermittent calorie deprivation (24 h), and Naive group (*n* = 32/genotype) received neither sweetened fat access nor intermittent calorie deprivation. Genotypes of mice were homozygous for the A allele (AA) or the variant G allele (GG) of *OPRM1*. **(A)** Intakes (Kcal) during the 30-min access period on *days 3* and *6*. **(B)** Comparison of *first binge* and *last binge* with representative caloric intake in the Restrict Binge and Binge groups. The black bars are Kcals derived from chow, whereas the white bars are the Kcals derived from sweetened fat. ** indicates *p* < 0.005 from first binge. **(C)** Total cumulative calories over the entire 6 weeks. ** indicates *p* < 0.005 from Naive group. **(D)** Final body weights after the 6 weeks. # indicates *p* < 0.05 from GG Binge and GG Naive groups.

Calorie intakes during 30-min intakes were different based on the intermittent access to sweetened fat (i.e., Restrict Binge and Binge groups) and intermittent calorie deprivation (i.e., Restrict Binge and Restrict). There were overall group [*F*(3, 233) = 526.7, *p* < 0.0001], time [*F*(11, 2,563) = 41.3, *p* < 0.0001], and group × time [*F*(33, 2,563) = 12.5, *p* < 0.0001] effects. *Post hoc* testing revealed an increase over time (i.e., escalation of intake) in the Restrict Binge and Binge groups (*p* < 0.005 for both); see Figure 1A. There were no genotype effects.

### Dietary Contribution of Calorie Intake During the 30-Min Access on First and Last Binge in Restrict Binge and Binge Groups

The increase in caloric intakes in the Restrict Binge and Binge groups resulted from the increase in sweetened fat intake. For the Restrict Binge and Binge groups, there was an effect for time [*F*(1, 60) = 64.0, *p* < 0.0001] and [*F*(1, 61) = 25.9, *p* < 0.00001], respectively. *Post hoc* testing revealed an increase in sweetened fat intake from the first to the last binge (*p* < 0.005 for both); see Figure 1B. There were no genotype effects.

### Cumulative Calorie Intakes Over the 6-Week Feeding Protocols

For cumulative total calorie intakes over the 6-week feeding schedule, there was a group effect [*F*(3, 247) = 43.9, *p* < 0.0005]. *Post hoc* testing revealed that there was a lower intake in Binge Restrict and Restrict groups (*p* < 0.005 for both) compared with the Naive group; see Figure 1C. There were no genotype effects.

### Body Weights Over the 6-Week Feeding Protocols

**Figure 2:**
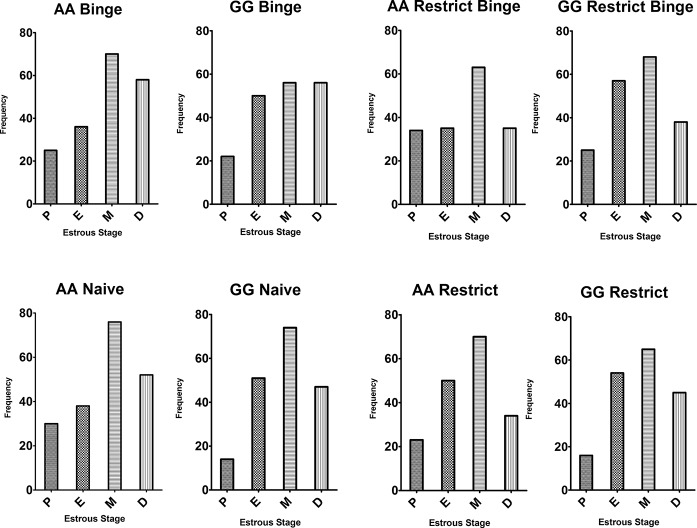
Estrous cycle frequency during the dietary-induced binge eating feeding protocol. Vaginal cytology was used to determine stage of estrous cycle. Data are from “binge” days (*days 3* and *6*) expressed as frequency or the number of occurrences of each stage over the 6-week feeding protocol. P, Proestrus; E, Estrus; M, Metestrus; D, Diestrus.

For body weight, there was a genotype × group effect [*F*(3, 247) = 4.69, *p* < 0.005]. *Post hoc* testing revealing that in the GG Restrict body weight was higher than AA Restrict (*p* < 0.05), and a similar trend of increased body weight was observed with the GG Restrict Binge compared with AA Restrict Binge (*p* = 0.06). There was also a time effect [*F*(11, 2,717) = 939.6, *p* < 0.0001] with an increase in body weight over the 6-week period in all groups. For the final body weights after the 6-week feeding protocol, there was a group effect [*F*(3, 247) = 4.8, *p* < 0.005], whereas a group × genotype effect approached significance [*F*(3, 247) = 2.6, *p* = 0.05]. Planned comparison revealed that GG Restrict had an increased body weight compared with GG Binge and GG Naive (*p* < 0.05 for both); see Figure 1D. The frequency of the stage of estrous was recorded 8 h prior to 30-min intakes throughout the 6-week period, most mice were either metestrus or diestrus; see Figure 2. These data demonstrate that the genotype does not influence the frequency of the stages of estrous over the 6-week feeding schedules.

### Calorie Intakes During the 30-Min Access Period During Acute Dosing of Lisdexamfetamine and Sibutramine

**Figure 3:**
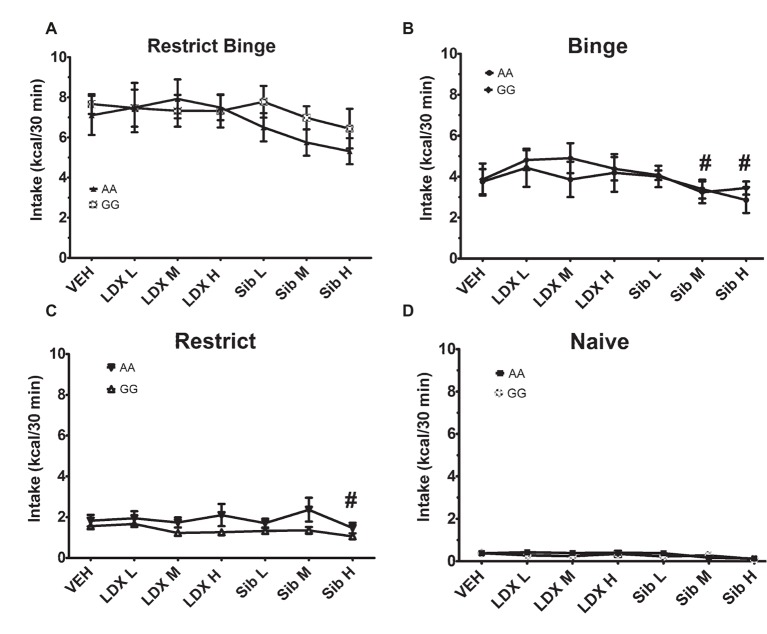
Intakes (Kcal) for 30-min access period on *day 6* for acute dosing following the 6-week dietary-induced feeding protocols. Data are expressed as mean ± SEM and are calorie intakes during the 30-min access periods. Each mouse was orally dosed with vehicle (VEH; water), lisdexamfetamine (LDX), and sibutramine (Sib). Dosing was performed with 1-week washout period. Mice were kept on the respective feeding protocol throughout (*n* = 7–8/genotype/group). Groups were **(A)** Restrict Binge, **(B)** Binge, **(C)** Restrict, **(D)** Naive. Lisdexamfetamine doses were 0.15 mg/kg (Lis L), 0.5 mg/kg (LDX M), and 1.5 mg/kg (LDX H). Sibutramine doses were 0.3 mg/kg (Sib L), 1 mg/kg (Sib M), and 3 mg/kg (Sib H). There were no genotype effects. For **(B)** and **(C)**, # indicates *p* < 0.05 from LDX L dose.

After the 6-week feeding protocols, mice were maintained on their respective feeding schedules and received ascending doses of vehicle, lisdexamfetamine, and sibutramine. Lisdexamfetamine and sibutramine were dosed in ascending fashion, and the order of whether lisdexamfetamine or sibutramine dosed first was random. For the 30-min intakes, there was an overall feeding group effect [*F*(3, 54) = 105.1, *p* < 0.0001] and overall treatment effect [*F*(6, 324) = 5.8, *p* < 0.00001]. For the overall feeding group effect, all groups were significantly different from each other (*p* < 0.0001). For the overall treatment effect, the sibutramine (3.0 mg/kg; Sib H) produced an overall reduction in 30-min intake compared with vehicle (*p* < 0.001) and all doses of lisdexamfetamine (*p* < 0.01 for all). Because all feeding groups were significantly different from each other, individual ANOVAs with repeated measures were performed for each feeding group to determine the genotype, treatment, and genotype × treatment effects. For the Binge group, there was a treatment effect [*F*(6, 84) = 3.16, *p* < 0.01]. Intakes following sibutramine (3.0 mg/kg; Sib H) and sibutramine (1.0 mg/kg; Sib M) were significantly reduced from lisdexamfetamine (0.15 mg/kg; Lis L; *p* < 0.05 for all); Figure 3B. For the Restrict group, there was a treatment effect [*F*(6, 84) = 2.8; *p* < 0.01]. Intakes following sibutramine (3.0 mg/kg; Sib H) were significantly reduced from lisdexamfetamine (0.15 mg/kg; Lis L; *p* < 0.05); Figure 3C. Neither the Restrict Binge nor Naive groups demonstrated significant effects; Figures 3A,D. There were also no genotype differences with treatments among the feeding groups.

### Calorie Intakes During the 30-Min Access Periods Throughout the Chronic (14-Day) Daily Dosing of Lisdexamfetamine and Sibutramine

**Figure 4:**
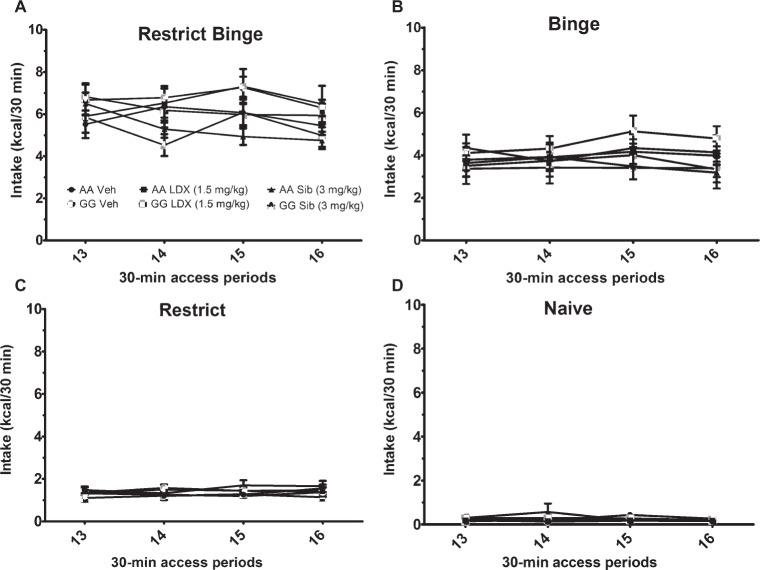
Intakes (Kcal) for 30-min access period on *days 3* and *6* during the chronic (14-day) daily dosing. Data are expressed as mean ± SEM. Mice (*n* = 8/genotype/treatment) were orally dosed with either vehicle (VEH; water), lisdexamfetamine (LDX), or sibutramine (Sib). Mice were exposed to the 6-week dietary-induced feeding protocols prior to dosing and were kept on the respective feeding protocol throughout. Groups were **(A)** Restrict Binge, **(B)** Binge, **(C)** Restrict, **(D)** Naive.

Following the 6-week feeding protocol, individual groups were dosed daily with sibutramine (3.0 mg/kg) and lisdexamfetamine (1.5 mg/kg) for 14 days. There were feeding group effects [*F*(3, 165) = 300.5, *p* < 0.0001] with all feeding groups being significantly different from each other (*p* < 0.0005). Individual two-way ANOVAs did not reveal significant effects of dosing treatments within the feeding groups; see Figure 4.

### Cumulative Calorie Intakes Throughout the Chronic (14-Day) Daily Dosing of Lisdexamfetamine and Sibutramine

**Figure 5:**
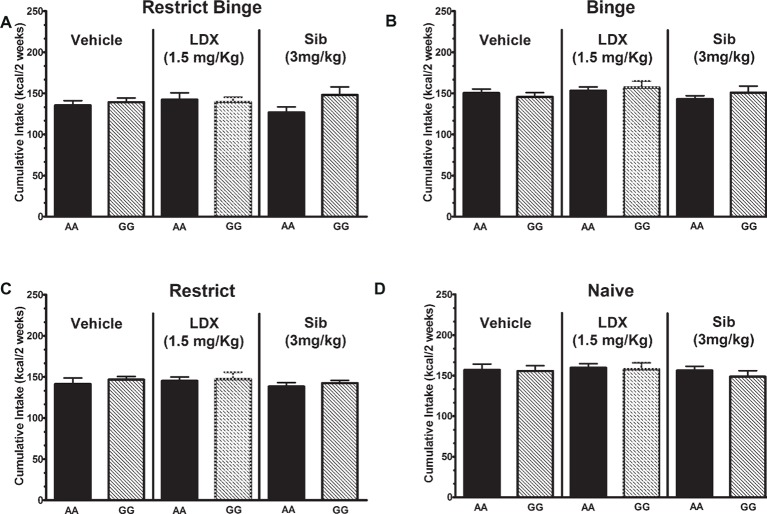
Cumulative intakes (Kcal) during the chronic (14-day) daily dosing. Data are expressed as mean ± SEM. Mice (*n* = 8/genotype/treatment) were orally dosed with either vehicle (VEH; water), lisdexamfetamine (LDX), or sibutramine (Sib). Mice were kept on the respective feeding protocol throughout. Groups were **(A)** Restrict Binge, **(B)** Binge, **(C)** Restrict, **(D)** Naive.

For cumulative calorie intakes over the 14-day treatments, there was a group effect [*F*(3, 168) = 8.3, *p* < 0.0005]. *Post hoc* testing revealed that there was a lower intake in Binge Restrict and Restrict groups (*p* < 0.005 for both) compared with the Naive group. Individual two-way ANOVAs did not reveal significant effects of dosing treatments within the feeding groups; see Figure 5.

### Body Weights Throughout the Chronic (14-Day) Daily Dosing of Lisdexamfetamine and Sibutramine

**Figure 6:**
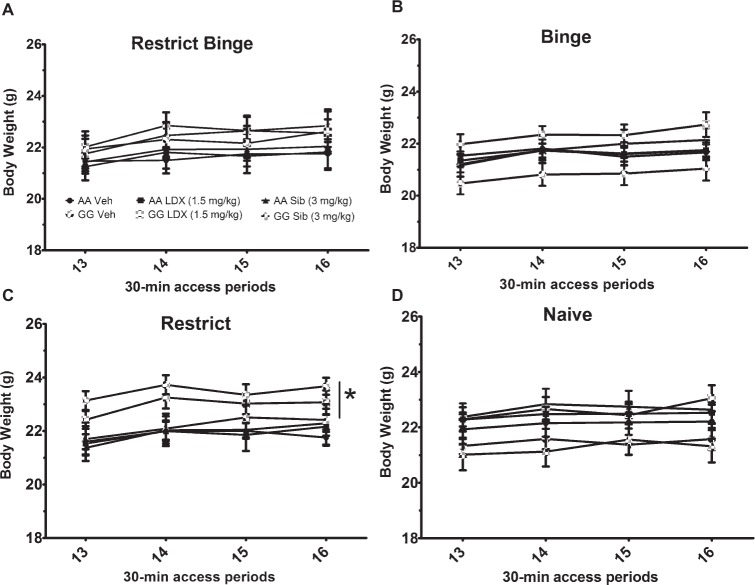
Body weights during the chronic (14-day) daily dosing. Data are expressed as mean ± SEM. Body weights were measured on *days 2* and *5* of the feeding schedules and corresponds to the 30-min access periods. Mice (*n* = 8/genotype/treatment) were orally dosed with either vehicle (VEH; water), lisdexamfetamine (Lis), or sibutramine (Sib). Mice were kept on the respective feeding protocol throughout. Groups were **(A)** Restrict Binge, **(B)** Binge, **(C)** Restrict, **(D)** Naive.* indicates *p* < 0.05 higher body weight in GG compared with AA.

For body weights over the 14-day treatment, there was a group effect [*F*(3, 168) = 2.75, *p* < 0.05]. *Post hoc* testing revealed that mice exposed to the Binge feeding protocol weighed less than the mice exposed to the Restrict feeding schedule (*p* < 0.05). There was also a genotype × group effect [*F*(3, 168) = 3.7, *p* < 0.05] with the GG Restrict having an increased body weight compared with AA Restrict (*p* < 0.05). There were also time [*F*(3, 504) = 75.0, *p* < 0.005], time × treatment [*F*(6, 504) = 4.2, *p* < 0.005], and time × genotype effects [*F*(3, 504) = 3.1, *p* < 0.05]. Both genotypes demonstrated an increase in body weight over the 14-day treatment (*p* < 0.005), see Figure 6.

## Discussion

The first objective of this study was to investigate whether binge-like eating behavior was differentially influenced by genotype in the A118G *OPRM1* murine model. The mice used in the present study were generated by a targeted homologous recombination in exon 1 of the *OPRM1* gene at the 112 position. Our mice were generated by similar methods used by others to generate a murine model of the A118G *OPRM1* polymorphism (Mague et al., 2009). Dietary-induced binge eating was defined by the amount of calories consumed in a short period of time (i.e., increased rate of eating) and the increased intake of the sweetened fat over time (i.e., escalating intake) (Corwin et al., 2011; Perello et al., 2014). Our findings in the present study indicate that female mice exposed to the Binge and Restrict Binge feeding schedules consumed excessive number of calories in a relatively short period of time and increased intake over the 6-week protocol, which was a result of the escalation in a highly palatable food (i.e., sweetened fat) intake from binge 1 to binge 12. The Restrict Binge feeding group had intermittent episodes of calorie restriction prior to the scheduled access to the sweetened fat to recapitulate the homeostatic drive (i.e., hunger) to overeat in the presence of a highly palatable food. In contrast, the Binge feeding group was not exposed to calorie restriction in order to recapitulate the hedonic drive to overeat (i.e., overeating when not calorie deprived). Taken together, these two feeding groups are subjected to the dietary conditions (i.e., prior dietary restriction and excess highly palatable food consumption) that influence binge eating behaviors in clinical populations (Association, 2013). Although these binge eating behaviors displayed by the mice do not capture the “loss of control” aspect of binge eating reported in humans, the limited access intermittent feeding schedule of a highly palatable food recapitulates the recurrent cyclical nature of binge eating (Bello and Hajnal, 2010). Our findings in the present study indicate that the AA or GG status of the 112 position of the *OPRM1* gene did not differentially influence binge-like eating. However, there were genotype-related differences in the Restrict feeding group. The Restrict group accounted for the intermittent schedule and exposed mice to twice weekly episodes of 24 h calorie deprivation prior to refeeding standard chow. These mice did not have repeated access to the sweetened fat. Over the 6-week period, the GG Restrict mice had a higher body weight than the GG Binge and GG Naive. During the chronic treatment conditions in the Restrict groups, there was an overall higher body weight in GG compared with AA mice. Notably, in a longitudinal 4-year familial study of mothers (*n* = 2,460) and children (*n* = 3,720), there was no association of the G allele with body mass index (BMI) or waist circumference (Hardman et al., 2014). It has been demonstrated, however, that GG mice show a reduction in hypothalamic gene expression *AVP* (arginine vasopressin) and *Gal* (galanin) (Collins et al., 2018). Additionally, it is possible, as our results suggest, that mice with the A112G variant have impairments in body weight compensation following repeated bouts of intermittent calorie deprivation. To our knowledge, the role of A118G polymorphism and dieting has not been explored. Our results would suggest that G allele has a protective effect against the weight loss associated with chronic calorie restriction.

The second objective of this study was to investigate whether there was a lisdexamfetamine and genotype interaction on the binge-like eating behavior. In this study, lisdexamfetamine was compared with the monoamine reuptake inhibitor, sibutramine. Originally, FDA approved in 1997 for the management of obesity (and withdrawn from the market in 2010), sibutramine has been demonstrated to be effective at reducing binge eating behaviors in BED subjects (Wilfley et al., 2008) and non-selectively reducing food (i.e., both highly palatable food intake and chow) intake in a rat model of binge eating (Vickers et al., 2015). In our study, we found that an acute sibutramine dose (3 mg/kg) resulted in an overall reduction of 30-min intakes compared with vehicle and lisdexamfetamine doses. However, in the Binge and Restrict groups, doses of sibutramine were only significantly different from lisdexamfetamine. In the 14-day chronic dosing, there were not any differences in body weight and food intakes with sibutramine (3 mg/kg/day) treatment. One reason for this discrepancy in the actions of sibutramine is that long-term sibutramine treatment has been shown to be more effective in obese vs. lean rodents and rather ineffective in body weight reduction in rodents fed a low-fat diet (Bush et al., 2006; Smith et al., 2011). In present study, the mice did not have an obese phenotype. Previous studies using female rats have demonstrated that lisdexamfetamine (0.1–1.5 mg/kg) dose dependently reduced chocolate intake in a limited access binge eating model (Vickers et al., 2015). In addition, lisdexamfetamine (0.8 mg/kg) attenuated the impulsive behaviors (i.e., delayed discounting task) and compulsive and preservative behaviors (i.e., punished responding model) displayed by binge eating female rats (Heal et al., 2016; Vickers et al., 2017). However, in the present study, we did not observe an influence of lisdexamfetamine (0.15, 0.5, 1.5 mg/kg) on dietary-induced binge eating in mice. There are several possible explanations. One consideration is that there are limited data on the use of lisdexamfetamine in mice. A study examining the potential dependency properties of lisdexamfetamine using mice revealed a conditioned place preference at an oral dose of 2.5 mg/kg, but not at an oral dose of 1.0 mg/kg (Yun et al., 2017). This suggests that a lisdexamfetamine dose greater than 1.5 mg/kg could have produced off-target reinforcing effects. As such, we used a lower dose range of lisdexamfetamine to uncover genotype-dependent differences without producing a ceiling effect in reducing binge-like eating. Another consideration is that lisdexamfetamine is a prodrug, which is dependent on the peptidase cleavage of lysine from the amphetamine moiety, and there are no comparative pharmacokinetics data available to suggest that cleavage of lysine is similar between rodent species. Therefore, the onset of action and pharmacokinetics of lisdexamfetamine were based on the rat data for the present studies.

Women are at a greater risk for developing an eating disorder (Association, 2013). Consequently, we used female mice in this study. In addition, sex-dependent differences have been observed in *A112G OPRM1* mice (Mague et al., 2009; Browne et al., 2017). Specifically, female GG mice demonstrate less withdrawal and morphine-pairing behaviors than AA females, whereas there were less apparent genotype-dependent differences in males (Mague et al., 2009; Browne et al., 2017). Additionally, previous studies have indicated that GG mice self-administer more heroin than AA mice (Zhang et al., 2015). At the lowest dose (0.0625 mg/kg), however, GG female mice demonstrated more operant responding (i.e., nose pokes) for heroin than AA females. In addition, there was also a greater heroin-induced striatal dopamine release in GG mice, compared with AA mice, and a greater mean dopamine release in female GG mice compared with male GG mice (Zhang et al., 2015).

Even though the association of the A118G *OPRM*1 variant with *specific* substances is debatable (Crist and Berrettini, 2014; Sloan et al., 2018), a collaborative meta-analysis (*n* = 28,689 subjects with European ancestry) indicated that the G allele had a protective effect on *general* substance dependence (OR = 0.90; 95% CI: 0.83–0.97) (Schwantes-An et al., 2016). The major finding of the present study was that the murine *A118G OPRM1* polymorphism did not have a differential influence on binge-like eating in female mice. Our mice had a guanine substituted at adenine region on 112 position exon 1 of *OPRM1.* This substitution strategy at this position of exon 1 has been shown to produce a similar reduction in *N*-glycosylation sites on the mu-opioid receptor protein (Huang et al., 2012). Another method for uncovering the functional role of the A118G polymorphism is to replace the mouse DNA sequence of exon 1 of *OPRM1* with the human DNA sequence of exon 1, which creates a chimera protein of the mu-opioid (Mahmoud et al., 2011; Freet et al., 2015). Nonetheless, binge-like eating or feeding responses have not been studied in the “humanized” A118G mouse models, and it is unknown whether there are differences in the humanized A118G mouse model. Therefore, our studies support the notion that there is no functional associate of the A118G *OPRM1* polymorphisms with binge eating or medications that suppress binge-like eating in rodents.

## Data Availability

The datasets generated for this study are available on request to the corresponding author.

## Author Contributions

NB conceived and designed the study, analyzed the data, wrote and edited the manuscript. BS drafted the manuscript and conducted the experiments. LY provided mice and assisted with designed and analyses. GG conducted the experiment and wrote part of the manuscript.

### Conflict of Interest Statement

The authors declare that the research was conducted in the absence of any commercial or financial relationships that could be construed as a potential conflict of interest.
